# The first intracellular loop is essential for the catalytic cycle of the human ABCG2 multidrug resistance transporter

**DOI:** 10.1002/1873-3468.13994

**Published:** 2020-11-21

**Authors:** Narakorn Khunweeraphong, Karl Kuchler

**Affiliations:** ^1^ Max Perutz Labs Vienna Center for Medical Biochemistry Campus Vienna Biocenter Medical University of Vienna Austria; ^2^ St. Anna Children’s Cancer Research Institute‐CCRI Vienna Austria

**Keywords:** ABCG2, anticancer drug resistance, ATP‐binding cassette transporter, catalytic cycle, transmission interface, transport mechanism

## Abstract

The human multidrug transporter ABCG2 is required for physiological detoxification and mediates anticancer drug resistance. Here, we identify pivotal residues in the first intracellular loop (ICL1), constituting an intrinsic part of the transmission interface. The architecture includes a triple helical bundle formed by the hot spot helix of the nucleotide‐binding domain, the elbow helix, and ICL1. We show here that the highly conserved ICL1 residues G462, Y463, and Y464 are essential for the proper cross talk of the closed nucleotide‐binding domain dimer with the transmembrane domains. Hence, ICL1 acts as a molecular spring, triggering the conformational switch of ABCG2 before substrate extrusion. These data suggest that the ABCG2 transmission interface may offer therapeutic options for the treatment of drug‐resistant malignancies.

## Graphical abstract

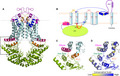

## Abbreviations

**ICL**, intracellular loop

**MDR**, multidrug resistance

**NBDs**, nucleotide‐binding domains

**PDR**, pleiotropic drug resistance

**THB**, triple helical bundle

**TMDs**, transmembrane domains

ABC transporters comprise one of the largest family of ubiquitous membrane transport proteins operating in all kingdoms of life as exporters and importers for a bewildering array of substrates [1]. Moreover, inborn genetic errors in human ABC genes cause prominent genetic diseases, including cystic fibrosis [2], gout [3], and severe disorders in lipoprotein homeostasis [4]. Importantly, ectopic overexpression of many ABC transporters is responsible for, or associated with, clinical multidrug resistance (MDR) to anticancer and anti‐infective drugs [5].

Eukaryotic ABC transporters consist of at least four separable functional domains: two highly conserved nucleotide‐binding domains (NBDs) and two transmembrane domains (TMDs), arranged in various configurations such a TMD‐NBD‐TMD‐NBD configuration in a single transporter [6, 7]. The human ABCome contains 48 genes encoding ABC proteins classified into seven subfamilies from ABCA to ABCG [8]. The ABCG subfamily has several remarkable features unique within the ABC superfamily. For example, ABCG2 and ABCG5/G8 encode half‐transporters, adopting a homo‐ or heterodimeric ‘reverse’ NBD‐TMD configuration, respectively [7, 9]. ABCG2 is a prototypic MDR transporter, mediating expulsion of hundreds of lipophilic molecules and toxins [10]. By contrast, ABCG5/G8 shows highly restricted sterol transport, protecting from unwanted toxicity of phytosterol compounds [11, 12]. Similarly, the closely related full‐length transporters ABCG1 and ABCG4 mediate cholesterol transport and are implicated lipoprotein metabolism [13, 14, 15]. Of note, mammalian ABCGs are the closest orthologues of yeast pleiotropic drug resistance (PDR) pumps, mediating antifungal MDR [16, 17], including Pdr5 and Cdr1, the best‐characterized fungal efflux transporters [18, 19, 20].

Of note, ABCG2 shares its promiscuous selectivity with the human P‐gp (ABCB1) and MRP1 (ABCC1) transporters, both of which are thought to cause clinical MDR (for recent reviews, see this special issue). However, the detrimental actions of ABCG2, P‐gp, and MRPs in cancer are balanced by their pivotal roles in physiological drug disposal, as they act as brothers in arms mediating detoxification across most epithelial barriers, ranging from placenta, GI tract to the blood–brain barrier [21, 22, 23, 24, 25].

Recent near‐atomic structures of ABC transporters, including P‐gp from various species [26, 27, 28, 29, 30, 31, 32] and human MRP1 [33, 34], facilitated efforts aimed at reaching in‐depth mechanistic understanding of catalytic cycles. Surprisingly, however, despite radically different physiological functions [35], MRPs and P‐gp share similar folds with the CFTR chloride channel [36, 37] or the sulfonylurea receptor [38]. By contrast, the first X‐ray atomic structure for the human ABCG5/G8 heterodimer [12], followed by cryo‐EM structures of ABCG2 in both inward‐ and outward‐facing conformations [39, 40, 41, 42], revealed a new and unique eukaryotic ABC fold. Furthermore, homology modeling approaches [43] and structure–function analysis [44, 45] verified the domain architectures of ABCG members, yet the molecular understanding about the catalytic cycles remains incomplete.

The ‘primordial’ alternating access model [46] is thought to apply to some ABC importers [47] as well as exporters [48, 49, 50]. The increasing numbers of atomic structures challenge the notion of a single unifying transport mechanism. NBDs certainly drive the catalytic cycle, but the dynamics of key domains and the cross talk between NBDs and TMDs remain unclear or at least matter of controversy, which could arise from ABC transporter‐specific mechanisms [6, 7, 63]. Hence, mechanistic similarities between diverse ABC proteins are likely confined to dimerization and ‘activation’ of NBDs that determine accessibility of substrate binding sites or the ability to undergo the conformational switch. The TMDs forming putative substrate translocation pores are more diverse in both sequence and architecture, as they must handle a broad spectrum of distinct substrates and inhibitors [64, 65]. Indeed, ABCG members have an importer‐like fold that is unique in the eukaryotic ABC family [12], supporting the notion of distinct mechanisms for ABCG members [56, 66], but also raising more questions than answers, certainly for the ABCG family [60].

In human ABCG2, the NBDs are in closer contact, subtending the transmission interface that connects NBDs with TMDs. The transmission interface in ABCG2 holds a ‘triple helical bundle’ (THB) formed by (a) the elbow helix, (b) the first intracellular loop (ICL1), also known as coupling helix [28], and (c) the hot spot helix or E‐helix [12]. Of note, the amphipathic elbow helix exposes the hydrophobic part to the lipid bilayer, while the hydrophilic side faces the NBD surface. This arrangement anchors and stabilizes ABCG2 to the inner bilayer leaflet, yet allows for rotational elbow helix movement [44]. Several residues in the THB are highly conserved across the ABCG family, and thus may engage the transmission interface during NBD‐TMD communication or facilitate the conformational switch [44, 45]. However, the exact functional roles of ICL1 and how it may operate after substrate recognitions remain enigmatic.

Here, we unravel crucial roles of highly conserved ICL1 residues. We show that a small kinked loop formed by G462, Y463, and Y464 is essential for ABCG2 function. G462 introduces a turn at the bottom center of ICL1, and this position requires a small apolar residue for proper folding and interaction with the NBD surface. Remarkably, the extremely conserved Y464 residue in the center of NBD‐elbow‐ICL1 cluster is also essential for ATPase activity. Importantly, Y464 engages in a conserved salt bridge to stabilize the transmission interface. Hence, the THB is a rigid architecture critical for the dynamics of NBD‐TMD interactions. The dynamics of the transmission interface is a vital part of the catalytic cycle, and ICL1 is acting as a molecular clutch that controls the inward–outward switch. These data pave the way for a complete understanding of the catalytic cycle used by the ABCG2 efflux pump. Importantly, our data suggest that ICL1 could be a feasible target for therapeutic intervention in case of drug‐refractory cancer malignancies.

## Materials and methods

### Chemicals and antibodies

All chemicals were of molecular biology grade from Sigma‐Aldrich (St. Louis, MO, USA), unless stated otherwise. Mitoxantrone, rhodamine 123, pheophorbide A, Ko143, potassium antimony (III) tartrate hydrate, adenosine 5′‐triphosphate disodium salt (ATP), ammonium molybdate, ascorbic acid, and ouabain octahydrate were from Sigma‐Aldrich. Poly‐ethyleneimine was from Polysciences Europe (Eppelheim, Germany). Sodium orthovanadate was from New England Biolabs (Frankfurt, Germany). G418 was from Santa Cruz Biotechnology (Dallax, TX, USA). Oligonucleotides for site‐directed mutagenesis were purchased from Eurofins (Munich, Germany). Monoclonal mouse anti‐ABCG2 (BXP‐21) was obtained from Santa Cruz Biotechnology (Santa Cruz, CA, USA). Rabbit anti‐β‐actin (D6A8) was purchased from Cell Signaling (Danvers, MA, USA). Phusion DNA polymerase and *DpnI* are from NEB (New England Biolabs, Ipswich, MA, USA).

### Plasmids constructions

All mutations were generated in vectors pcDNA3.1(‐)‐hABCG2 or pEGFPC1‐hABCG2 plasmids as templates. Side‐directed mutagenesis was applied to create mutants using Phusion DNA polymerase (NEB) according to the manufacture’s manual, following by *DpnI* digestion to eliminate DNA template before transforming into *Escherichia coli* (DH5α) for plasmid preparation and verification by DNA sequencing. The primers used in this study are indicated in Table S1.

### Mammalian cell culture and transfection

Human embryonic kidney cells (HEK293) were cultured in Dulbecco’s modified Eagle’s medium (DMEM; Life Technologies, Rockville, MD, USA), supplemented with 5% (v/v) FBS at 37 °C, 5% CO_2_ with humidity. HEK293 cells were seeded onto a 6‐well plate 1 day prior to transfection using self‐made Poly‐ethyleneimine (Polysciences Inc.) with 2 µg of plasmids in 100 μL of Opti‐MEM (Life Technologies). Two days after transfection, cells were subjected for subsequent experiments.

### Immunodetection

Cells were harvested after trypsin digestion, followed by a washing with ice‐cold PBS (pH 7.4). Cell pellets were then lysed in ice‐cold lysis buffer containing 50 mm Tris (pH 8.0), 120 mm NaCl, 1 mm EDTA, 2% Triton X‐100, and freshly added protease inhibitor cocktail (Halt Protease and Phosphatase inhibitor Cocktail; Thermo Scientific, Rockford, IL, USA). Cell debris was removed by centrifugation at 1200 ***g*** for 2 min at 4 °C. The supernatants were collected and mixed with Laemmli sample buffer in the presence of absence of 100 mm DTT before subjected to SDS/PAGE. Western blot was performed according to standard protocols. After electro‐transferred onto nitrocellulose (GE Healthcare Life sciences, Freiburg, Germany), membranes were blocked in blocking solution, 5% BSA in TBST buffer (Tris‐buffered saline containing 0.1% Tween‐20), at room temperature for 1 h. The primary mouse anti‐ABCG2 antibody (BXP‐21) (Santa Cruz Biotechnology, CA) or rabbit anti‐β‐actin (D6A8) (Cell Signaling) was added at dilutions of 1 : 500 and 1 : 1000, respectively. After incubation at 4 °C for overnight, the membranes were washed three times with TBST for 15 min. Then, add the IRDye® 800CW secondary antibodies (LI‐COR Biosciences, Homburg, Germany) against mouse (for anti‐ABCG2) or rabbit (for anti‐β‐actin) (at dilution 1 : 10 000 in TBST) and incubated at room temperature for 1 h. The signals were detected with the 800 channel of the Odyssey Imaging Systems and quantified by using image studio software version 2.1 (LI‐COR® Biosciences).

### ABCG2 transport assays

Transport activity of ABCG2 variants was measured by flow cytometry using fluorescent compound substrate, mitoxantrone or rhodamine 123 or pheophorbide A, in the presence or absence of ABCG2‐specific inhibitor, Ko143. Transfected cells were harvested by trypsin digestion and collected in DMEM (containing 5% FBS), followed by washing with ice‐cold PBS. The cell pellets containing 10^5^ cells per reaction were resuspended in HPMI buffer (10 mm Hepes, 120 mm NaCl, 5 mm KCl, 400 µm MgCl_2_, 40 µm CaCl_2_, 10 mm NaHCO_3_, 10 mm glucose, 5 mm Na_2_HPO_4_, pH 7.4). Cells were pre‐incubated with 1 µm Ko143 at 37 °C for 5 min before adding substrate to a final concentration, 7 µm mitoxantrone, or 0.5 µm rhodamine 123 or 1 µm pheophorbide A, then incubated at 37 °C for 20 min. The reactions were stopped by placing the tube into ice‐cold water, followed by adding ice‐cold PBS 500 μL and centrifuged at 600 ***g*** for 2 min at 4 °C. Supernatant was removed, and the cell pellets were resuspended in ice‐cold PBS for analysis using the BD FACSCalibur (Becton Dickinson, San Diego, CA, USA). Intracellular mitoxantrone or pheophorbide A was determined with FL3 at excitation and emission wavelength at 488 and 670 nm, while rhodamine 123 was measured with FL1 at excitation and emission wavelength at 488 and 534 nm, respectively. The viable cell population was gated, and 10^4^ cells were collected for each data point. Data were analyzed using the flowjo Software Inc. (Stanford University). The fluorescence intensity was corrected from unstained cells as a background. Signals were normalized to the activity in the presence of Ko143 inhibition, which was set to 100% inhibition. The results were represented as percentage of activity relative to wild‐type (WT) ABCG2.

### Membrane protein preparation

HEK293 cells expressing ABCG2 variants were cultured in 10 cm dishes. Cells were washed with ice‐cold PBS twice prior to harvesting. Cells pellets were resuspended in ice‐cold TMEP buffer (50 mm Tris pH 7.0, 50 mm Mannitol, 2 mm EGTA, and protease inhibitor cocktail) and lysed by passing the suspension through a 27‐gauge needle using a syringe for 20 times. Debris was removed by centrifugation at 500 ***g*** for 10 min; mitochondria protein was sedimented by centrifugation at 1200 ***g*** for 5 min. Supernatants were collected prior to ultracentrifugation at 100 000 ***g*** for 60 min. All procedures were always performed at 4 °C. Protein concentrations were measured by Bradford assays. The membrane proteins extracts were adjusted at 2 mg·mL^−1^ and stored at −80 °C.

### ATPase assays

The vanadate‐sensitive ATPase activity of ABCG2 was examined using membrane protein preparation from HEK293 cells. In brief, 5 µg of membrane protein was pre‐incubated in ATPase assay buffer (50 mm MOPS (3‐(N‐morpholino)‐propane‐sulfonic acid), 50 mm KCl, 0.5 mm EGTA, 5 mm NaN_3_, 2.5 mm DTT, 1 mm Ouabain, pH 7.0) at 37 °C for 10 min, in the presence or absence of 100 µm Na_3_VO_4_ (sodium orthovanadate). The reaction was started by addition of 4 mm of ATP/Mg^2+^ and incubated at 37 °C for 30 min. The reactions were stopped by adding 40 µL of 5% SDS. Then, 100 µL of color reagent containing 3.33% (v/v) H_2_SO_4_, 0.48% (w/v), ammonium molybdate, 0.006% (w/v), antimony potassium tartrate, 5.7% (v/v) acetic acid, and 0.24% (w/v) ascorbic acid (freshly prepared) was added and incubated at room temperature for 30 min. The signal of released inorganic phosphate was measured at the wavelength 750 nm using microplate reader (VICTOR Nivo; PerkinElmer, Turku, Finland). An SDS‐treated sample was used as a background. The vanadate‐sensitive ATPase activity was calculated by subtracting from vanadate‐treated sample. Data were represented as relative to WT.

### Confocal microscopy

HEK293 cells were seeded onto Ibidi glass chamber 8‐well plates. After culture for overnight, cells were transient transfected with plasmid using Poly‐ethyleneimine in Opti‐MEM. Two days after transfection, removed culture medium from the wells then washed with ice‐cold PBS and subsequently fixed with 4% formaldehyde in PBS at room temperature for 10 min. Cells were washed three times with PBS. Nuclei were stained with 5 µg·mL^−1^ DAPI (4′,6‐diamidino‐2‐phenylindole) in PBS at room temperature for 10 min. After washing cells with cold PBS three times, cells were incubated in 100 mm glycine for 15 min at room temperature. Samples were protected from light and continued for imaging with a Zeiss LSM700 confocal microscope using zen 2012 as analysis software (Zeiss, Oberkochen, Germany).

### Data and statistical analysis

All values in this study were represented as mean with SEM, unless stated otherwise. All *in vitro* experiments were performed at least three independent assays. Figures and statistical analyses were generated by GraphPad Software Inc. (San Diego, CA, USA) prism version 6.00. The molecular visualizations were performed using vmd, v1.9.2 (University of Illinois at Urbana‐Champaign, Urbana, IL, USA) and pymol, v1.8.4 (Schrödinger, LLC., New York, NY, USA).

## Results

### The ABCG2 transmission interface is a triple helical bundle

**Fig. 1:**
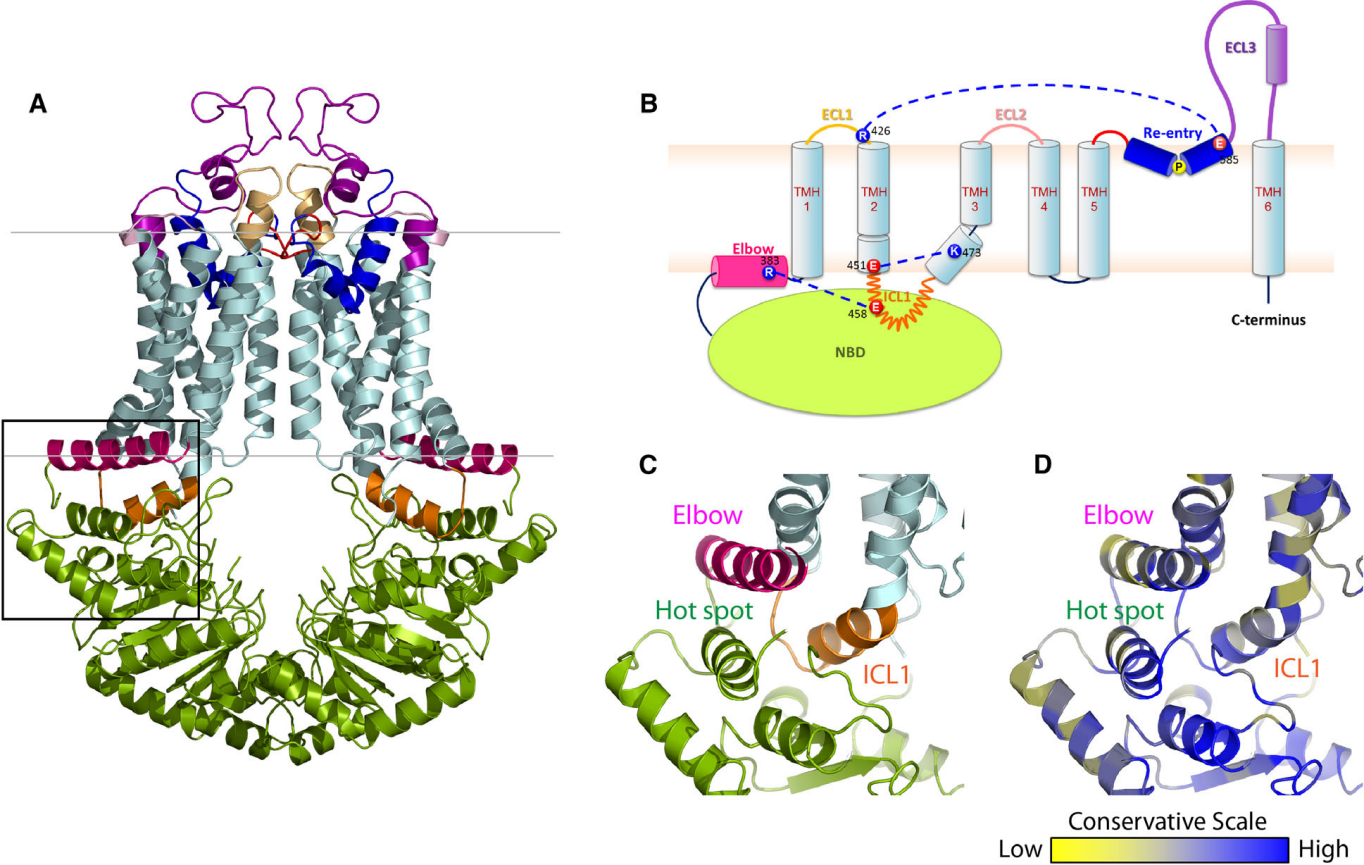
The structural organization of human ABCG2. (A) Inward‐facing configuration of the homodimeric ABCG2 transporter in the ATP‐free state (PDB ID: 6ETI). The black box delimits the THB cluster of three conserved helices at the transmission interface, NBD (green), elbow helix (pink), and first ICL (orange). Transmembrane‐spanning helices (light blue), extracellular loop 1 (yellow), re‐entry helix (dark blue), extracellular loop 3 (purple). (B) Membrane topology of ABCG2 half transporter indicating the salt bridge interactions at both membrane interfaces (blue dotted lines). Colors are as in panel (A). (C) Zoom‐in the black box of (A) shows adjacent structural arrangement of three important domains: NBD, elbow helix, and ICL1 at transmission interface. (D) The conservation analysis of (C) is given as color‐gradient: low conservation (yellow) to high conservation (blue).

The cryo‐EM structures of human ABCG2 in both inward‐ and outward‐facing conformations suggest a distinct fold resembling an importer rather than an exporter (Fig. 1A). Of note, mammalian ABCGs and fungal PDR pumps share conserved regions required for ATPase activity and efflux function, including A‐loop, Walker A and B, Q‐loop, mutational hot spot helix, signature loop, Pro‐loop, D‐loop, and H‐loop, respectively (Fig. S1A). Likewise, motifs in the TMDs, including elbow helix, TMH1‐TMH6, ICL1, hydrophobic valve, re‐entry helix, large extracellular loop 3, and short C terminus (Fig. S1B), are conserved. Moreover, two unique helical domains, the elbow helix and the re‐entry helix, are present at membrane interfaces, and these regions are stabilized by the salt bridge interactions (Fig. 1B).

**Fig. 2:**
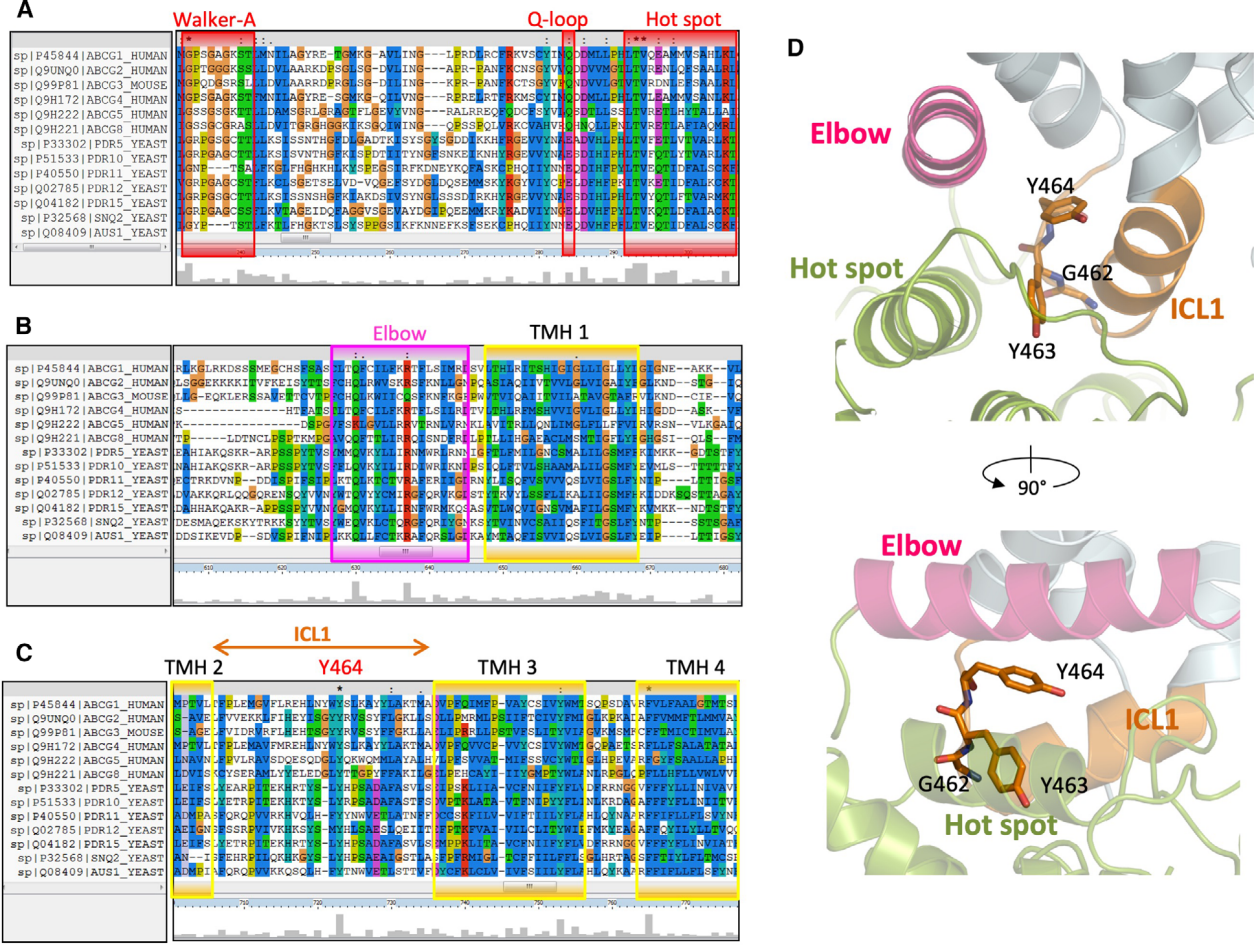
Sequence alignment of mammalian ABCGs and the first half of yeast PDRs. Multiple sequence alignment was analyzed using ClustalX2. Conserved residues are highlighted with the conservation scale as the height of gray bars at the bottom of each residue. (A) Conservative regions of Walker A, Q‐loop, and hot spot helix in the NBD are in red boxes. (B) Conserved regions of elbow helix (pink) and transmembrane helix 1(yellow). (C) Conserved regions in transmembrane helices 2, 3, and 4 (yellow). The positions of conserved residues glycine 462, tyrosine 463, and tyrosine 464 of human ABCG2 are marked in the first ICL among ABCGs family proteins. (D) Zoom‐in side views show side chains as sticks of three conserved residues in ICL1, G462, Y463, and Y464, respectively. NBD (green), elbow helix (pink), ICL1 (orange), transmembrane helices (light blue).

The transmission interface of eukaryotic ABC transporters is essential for the NBD‐TMD communication, inducing the conformational switch for substrate translocation through the transport channel [61, 68, 69]. In ABCG2, the transmission interface has a unique architecture, containing three highly conserved helices, the hot spot helix as part of the NBD, the elbow helix, and the ICL1, all of which arrange in a ‘THB’. Key residues of the THB as well as its folding are highly conserved in the ABCG and PDR family (Fig. 1C,D). The inward‐ and outward‐facing states hold a similar fold of THB, with a groove or socket‐like structure touching upon the upper part of the NBD dimer (Fig. 1A,C). This groove is formed by the conserved Q‐loop (Q126), and part of the highly conserved mutational hot spot helix (T135 to A149), providing a docking base for the interaction with ICL1 that connects TMH2 and TMH3 (Fig. 1B,C). The parallel mutational hot spot helix shares an interface with the elbow helix (Fig. 1C). This region contains several important residues for ABCG2 folding and function (Fig. 2A), including the Q141K polymorphic site linked to gout [3, 67]. The elbow helix (F373 to N391) connects the NBD with TMH1, and is located along the inner bilayer leaflet, with the hydrophobic side facing toward the membrane, and the hydrophilic side to the cytoplasm. The elbow helix forms a salt bridge with the ICL1 and thus anchors ABCG2 at the inner membrane aspect (Figs 1B and 2B). The first ICL1 of human ABCG2 is rather short when compared to other ABCB family transporters [39, 41, 42]. ICL1 stretches from residue E451 to R465, connecting TMH2 and TMH3. ICL1 is a conserved coupling helix [44, 54, 61, 68], engaging the upper part of the intramolecular NBD dimer and the elbow helix in the THB cluster (Fig. 1C). Remarkably, the ICL1 holds an extremely conserved domain formed by a kinked G462‐Y463‐Y464 stretch (Fig. 2D). Hence, we reasoned that the GYY stretch in ICL1 within the THB may be critical for the ABCG2 catalytic cycle.

### G462 in ICL1 provides a flexible u‐turn loop bridging the NBD dimer with the TMDs

**Fig. 3:**
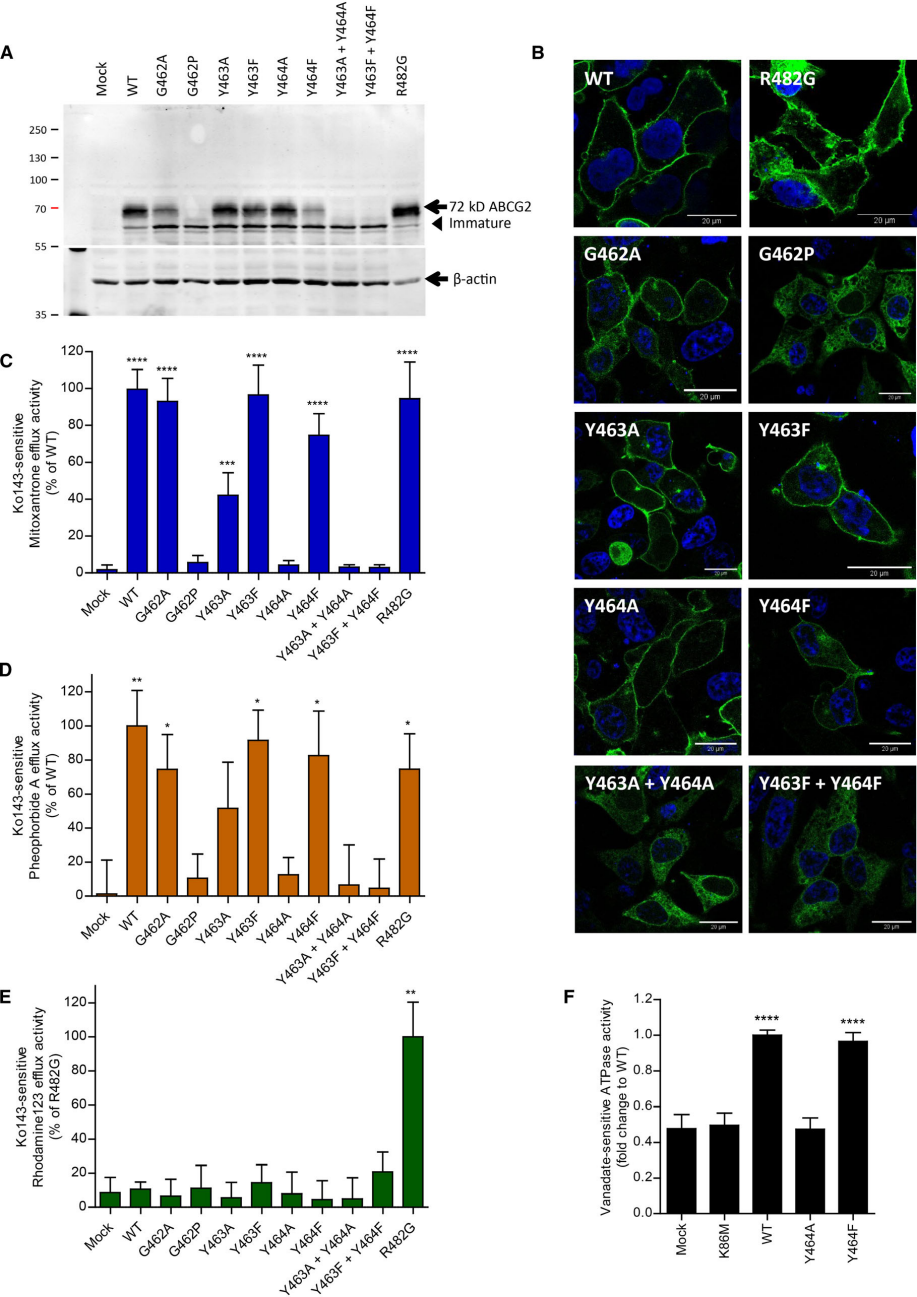
ICL1 contains essential residues. Three conserved residues located in the first ICL were subjected to mutational analysis, followed by functional expression in HEK293 cells. (A) Immunodetection of ABCG2 variants after transient transfection into HEK293 cells for 2 days using the monoclonal anti‐ABCG2 (BXP‐21) antibody. Mature WT protein migrates at ~ 72 kDa, whereas immature unglycosylated ABCG2 migrates just below. β‐actin was used as an internal loading control. (B) Membrane localization of GFP‐tagged ABCG2 variants visualized by confocal microscopy to detect GFP‐ABCG2 mutants (green). Nuclear DNA was stained with DAPI (blue). Microscopy data are from duplicate experiments. Scale bar in microscopy images corresponds to 20 μm. (C) Mitoxantrone efflux, (D) pheophorbide A efflux, and (E) rhodamine 123 efflux, in transfected HEK293 cells after incubation at 37 °C for 20 min in the presence and absence of the specific ABCG2 inhibitor, Ko143. Ko143‐sensitive efflux is represented as percentage relative to WT or R482G. Data are from several independent experiments (*n* = 3–8). (F) Vanadate‐sensitive ATPase activity of ABCG2 variants in transfected HEK293 membrane proteins. Vanadate‐sensitive ATPase activity is expressed as a fold change relative to the WT control. Data are from five independent experiments (*n* = 5) derived from three batches of membrane preparations. The ATPase‐dead mutant, K86M, was used as an additional control. All data are shown as mean and SEM. ****P < 0.0001, *** *P* < 0.001, ** *P* < 0.01 and * *P* < 0.1 vs. empty plasmid transfected HEK293 (mock).

The three conserved ICL1 residues, G462, Y463, and Y464 (Fig. 2C,D), are located after the coupling helix motif, with their side chains facing the core of the THB (Fig. 2C,D). Thus, we subjected the GYY domain to extensive mutational analysis by substituting each residue with alanine, proline, and phenylalanine. Moreover, we generated several double mutants, as well as GFP‐tagged versions. The ABCG2 mutant variants were expressed in HEK293 cells for functional analysis, including protein levels (Figs 3A and S2), subcellular localization (Fig. 3B), mitoxantrone transport assays in the presence or absence of the ABCG2‐specific inhibitor Ko143 (Fig. 3C), and ATPase activities (Fig. 3F). Further, we also included the ABCG2 substrates pheophorbide A (ABCG2‐WT‐specific substrate) and rhodamine 123 (R482G mutant‐specific substrate) in efflux assays (Fig. 3D,E). Finally, the R482G variant was included as a substrate‐specific control. R482G showed normal protein expression as assessed by rhodamine 123 efflux (Figs 3A‐E and S2). The K86M variant was used as a control for an ABCG2 ATPase‐dead mutant (Fig. 3F).

G462 introduces a flexible kink to create a turned loop at the bottom of the ICL1, touching the top of the NBD surface (Fig. 2D). Most mammalian ABCGs share this residue, though it disappeared in the full‐size PDR transporters family (Fig. 2C). Hence, we substituted G462 with alanine (G462A) or proline (G462P) to test their functional relevance. Alanine substitution in G462A mutant slightly reduced mature protein expression, membrane localization, and transport function, as quantified by mitoxantrone and pheophorbide A efflux. G462A did not alter substrate specificity as indicated by rhodamine 123 efflux (Figs 3A‐E and S2). Remarkably, and by sharp contrast, the G462P variant abrogated mature ABCG2 protein levels as well as drug transport. Immature ABCG2 protein was still observed though, but it accumulated intracellularly in exocytic secretory compartments (Figs 3A‐E and S2). The results indicated that G462 is essential for ABCG2 folding. However, a rigid kink introduced by proline disrupted both ABCG2 targeting and function. These data show that ICL1 not only requires a kinked structure, but also needs some flexibility perhaps to allow for the dynamics of the conformational switch during the catalytic cycle.

### The aromatic ring of Y463 is required for the full transport activity of ABCG2

Y463 is among highly conserved residues in the ABCG family (Fig. 2C); it turns the aromatic side chain into the middle of the interface between the mutational hot spot helix and the coupling helix (Fig. 2C). To determine the importance of Y463, we changed Y463 to alanine and phenylalanine to obtain the Y463A and Y463F variants. Both mutant variants showed normal protein levels at the plasma membrane (Fig. 3A,B). In addition, Y463F showed normal mitoxantrone and pheophorbide A efflux. Strikingly though, the loss of aromatic side chain in Y463A severely impaired both mitoxantrone and pheophorbide A efflux to about 50% when compared to the WT control, without altering substrate specificity (Fig. 3C‐E). These data suggest that Y463 is critical for function, indicating that it may be adding stability to the THB during the transport cycle.

### The conserved Y464 in the ICL1 is essential for ABCG2 function and the catalytic cycle

Importantly, Y464 within ICL1 is the most highly conserved residue in all mammalian ABCGs as well as in yeast PDRs, implying its pivotal role in ABCG family transporters (Fig. 2C,D). The cryo‐EM structures of ABCG2 in both inward‐ and outward‐facing conformations [39, 40, 41, 42] indicate that the side chain of Y464 is located in the middle of the THB, sharing its conserved interface with the elbow helix and the coupling helix. The Y464 side chain points into the core of the transporter toward the other ABCG2 half molecule (Fig. 2D). We therefore substituted Y464 by alanine or phenylalanine to construct the Y464A and Y464F variants. Surprisingly, even the phenylalanine substitution in Y464F was not well tolerated, since it diminished mature ABCG2 surface levels by ~ 50% (Fig. 3A,B). By contrast, a small aliphatic residue such as in Y464A still allowed for normal ABCG2 surface trafficking (Fig. 3A,B). However, the Y464A substitution fully destroyed efflux activity for all ABCG2 substrates (mitoxantrone, pheophorbide A, and rhodamine 123) tested (Fig. 3C‐E). Notably, the Y464F variant showed impaired efflux activity of about 25%, as well as reduced mature surface protein levels (Fig. 3A,B), though without altering substrate specificity (Fig. 3C‐E). Most strikingly, the Y464A mutant underwent normal surface trafficking (Fig. 3A,B), but showed completely abrogated drug efflux (Fig. 3C‐E).

We further scrutinized the defects to reveal whether or not it was related to defects in the catalytic cycle. Hence, we quantified vanadate‐sensitive ATPase activities of all variants, along with the kinase‐dead mutant K86M as a negative control. As expected, the K86M mutant was incompetent for ATP hydrolysis when compared to the mock control. WT ABCG2 exhibited a 2‐fold higher ATPase activity than the negative controls (mock and K86M), which was very similar to the Y464F ATPase activity. Strikingly, the Y464A variant lost the ability to consume ATP, indicating that the aromatic ring of the invariant Y464 is pivotal for ATP hydrolysis in ABCG proteins (Fig. 3F). To test whether or not the neighboring residues Y463 and Y464 can compensate for each other, we also generated the Y463A Y464A and Y463F Y464F double mutant variants. Notably, both double mutants showed abrogated surface protein levels and impaired efflux, since only immature, unglycosylated ABCG2 accumulated intracellularly (Fig. 3A‐E). Taken together, these data strongly suggest that the aromatic rings, and in particular the hydroxyl groups (‐OH) of the Y464 and Y463 residues, are critical for ABCG2 folding and function.

### Y464 establishes a rigid architecture in the THB through a salt bridge interaction

**Fig. 4:**
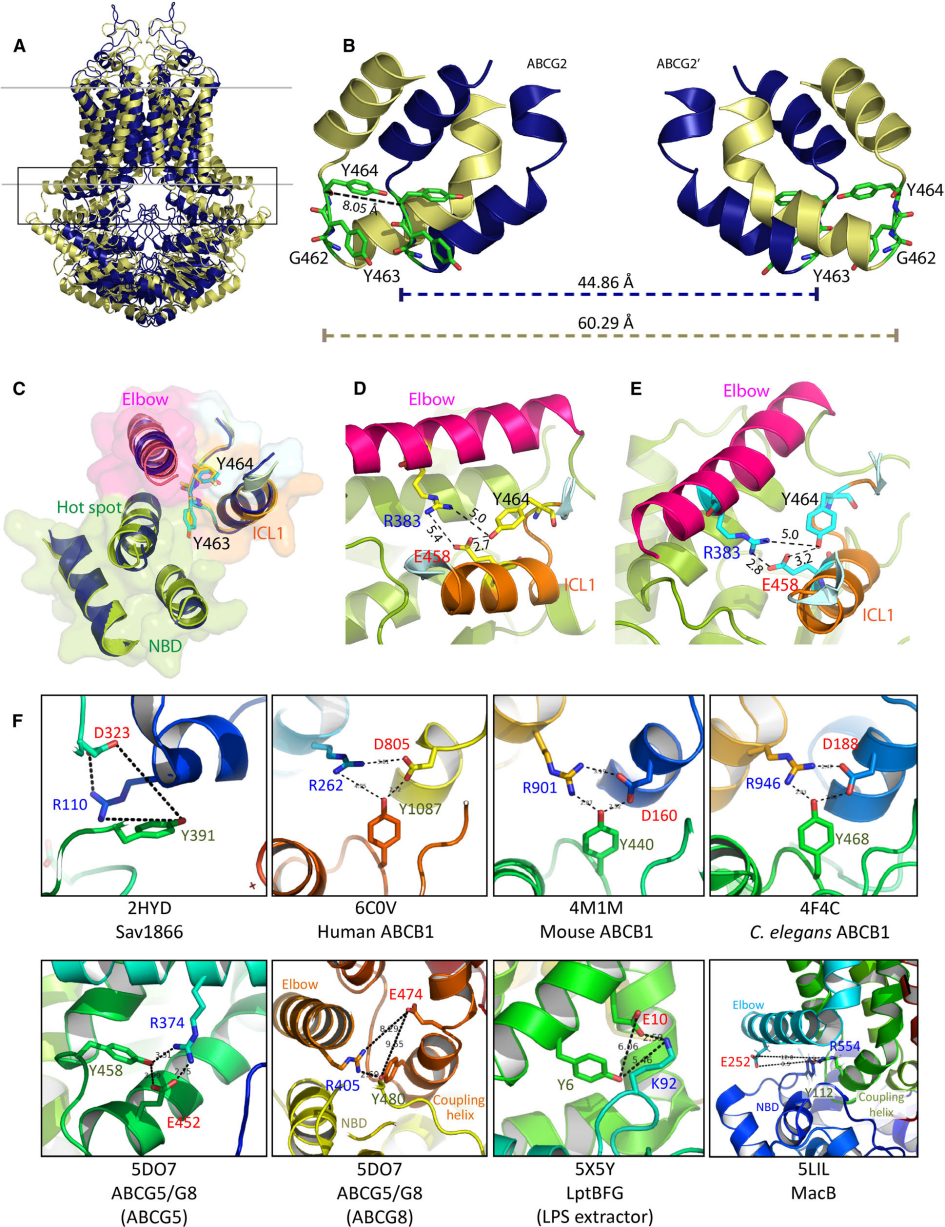
Restricted motion stabilizes ABCG2 and controls the transmission interface. (A) Overlay of ABCG2 conformations between inward (pale yellow, PDB ID: 6ETI)‐ and outward‐facing states (dark blue, PDB ID: 6HBU). The black box indicates the degree of movement at the transmission interface during the cycle. (B) The zoom‐in side view of the black box of (A) shows the superimposition of ICL1 in homodimeric ABCG2 between ATP‐free (pale yellow) and ATP‐bound (dark blue). Three conserved residues (G462, Y463, and Y464) are shown as green sticks. Dotted lines indicate the distance in Ångström (Å). (C) Superimposition of five elements forming a cluster at the inner membrane interface between inward (color ribbon)‐ and outward‐facing (dark blue ribbon) states. Transparent surfaces indicate NBD (green), elbow (pink), and ICL1 (orange). The side chains from inward‐facing (yellow sticks) and outward‐facing state (blue sticks) are shown. (D) The inward‐facing and (E) outward‐facing states with the distances of three conserved residues in the elbow helix (pink) and in ICL1 (orange) as dotted lines in Å. Residues are indicated as yellow (ATP‐free state) and pale blue sticks (ATP‐bound state). (F) Zoom‐in structures suggest engagement of highly conserved tyrosines in salt bridges of transmission interfaces found in Sav1866 (PDB ID: 2HYD), human ABCB1 (PDB ID: 6COV), mouse ABCB1 (PDB ID: 4M1M), *C. elegans* ABCB1 (PDB ID: 4F4C), human ABCG5/ABCG8 (PDB ID: 5DO7), LPS exporter (PDB ID: 5X5Y), and MacB (PDB ID: 5LIL).

ATP binding triggers NBD dimerization and thus imposes structural changes through the transmission interface. The superimposition of both inward‐ and outward‐facing states suggests dynamic motions of the transmission interface preceding the conformational switch (Fig. 4A). To assess a role for Y464 in the THB network, we inspected the dynamics of movements during the transport cycle using the inward (PDB ID: 6ETI)‐ and outward‐facing ABCG2 structures (PDB ID: 6HBU) (Fig. 4). The backbone or α‐carbon of Y464 moves toward the center core by around 8 Å. The distance of both Y464 residues between the ABCG2 monomers was 60.29 Å in the inward‐facing conformation vs. 44.86 Å in the outward‐facing state (Fig. 4B). Interestingly, however, the structural arrangement of the THB remained static, as evident from matching superimpositions of both conformations (Fig. 4C). Hence, the aromatic ring of Y464 appeared almost fixed in the middle of the THB (Fig. 4D,E). This observation implies that Y464 may limit the flexibility of the THB in the transmission interface.

The NBD‐TMD cross talk mediated by the transmission interface is a key to regulating ATP hydrolysis and the conformational switch that drives substrate translocation through the valve in the membrane core [45]. We reasoned that an additional salt bridge interaction through Y464 would aid the coupling of the transmission interface by conferring structural rigidity, yet allowing for a ‘leverage’ or clutch effect driving the conformational switch of ABCG2. Indeed, the transmission interface is stabilized by a conserved salt bridge between the R383 of the elbow helix and E458 in ICL1 [44]. Since Y464 holds its side chain in close proximity to the conserved salt bridge between the elbow helix and ICL1, we propose that Y464 may also interact with the conserved salt bridge R383‐E458 to further stabilize the fold and to facilitate catalytic cycle (Fig. 4D,E).

To compare the possibility of tyrosine engagement into salt bridge at transmission interface of other ABC transporters, we checked for possible salt bridges at transmission interface in other ABC exporters. Interestingly, although many ABC transporters have a rather unique fold at the transmission interface, the tyrosine residue was mostly located at the center hub, very close to a putative salt bridge in this region in distinct exporter subfamilies. For example, a tyrosine in this position is present in diverse ABCB family exporters (bacterial Sav1866, human ABCB1, mouse ABCB1, *Caenorhabditis elegans* ABCB1), ABCG exporters (human ABCG5/ABCG8), LPS extractor (bacterial LptBFG), and tripartite antibiotic exporter (bacterial MacB) (Fig. 4F). The vastly different substrate specificities of these distinct ABC exporter families strongly suggest that this highly conserved tyrosine establishes a key mechanical element, which is essential for driving the conformational switch rather than for substrate specificity of ABCG2.

## Discussion

**Fig. 5:**
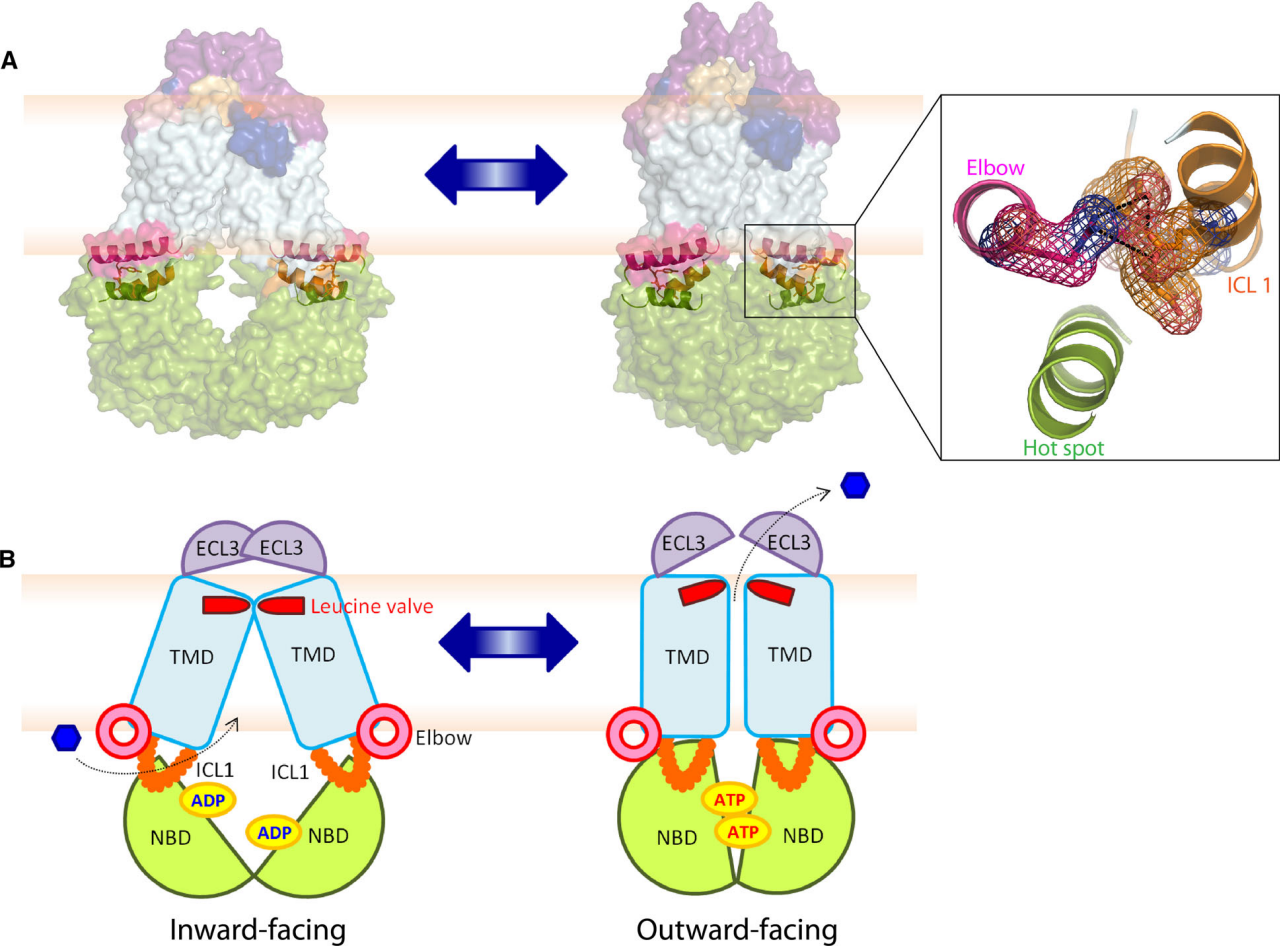
A rigid THB cluster in the transmission interface is essential for the conformational switch of human ABCG2. The structural models of homodimeric ABCG2 during the catalytic cycle between inward (left panel, PDB ID: 6ETI)‐ and outward‐facing states (right panel, PDB ID: 6HBU) are shown in the transparent space‐filling modes (A) and in hypothetical cartoon models (B). The THB clusters of conserved helices (NBD‐elbow‐ICL1) are shown. The structures show NBD (green), elbow helix (pink), TMD (light blue), ICL1 (orange), and ECL3 (purple).

The recent X‐ray crystal and cryo‐EM structures of the ABCG family members such as ABCG5/G8 [12] and ABCG2 [39, 40, 41, 42] facilitated homology modeling approaches [43, 44], as well as molecular structure–function approaches [44, 83], all in all unraveling mechanisms underlying the catalytic cycle of ABCG‐mediated transport. Remarkably, ABCG2, although resembling more closely an importer, is the hallmark MDR exporter able to handle hundreds of xenobiotic compounds in physiological detoxification [84, 85, 86, 87], as well as many anticancer drugs in tumor therapy [22, 88, 89, 90]. ABCG family members show a remarkable domain architecture, with unique elements only present in ABCG transporters [12, 39, 42]. ABCG2 operates like a peristaltic pump, where substrate export and release are tightly controlled by a hydrophobic valve in the transporter core and by a compact extracellular roof residing on top of the membrane, respectively [45]. Interestingly, the putative drug translocation channel in the core connects a large intracellular, ‘drug‐trapping’ cavity with a smaller outer, drug‐releasing cavity. Both cavities are communicating via the valve and undergo dynamic changes in shape and volume during drug transport which is driven by a conformational switch [41, 42, 44, 45]. Of note, the mechanical roles of intracellular domains such as the transmission interface in the conformational switch have remained unclear, although a tight coupling must exist to allow for the switch (Fig. 5).

Here, we show that the transmission interface contains a THB, which is essential for the catalytic cycle. The THB resides between the TMDs and the NBD surface, holding the NBD dimer in proximity to the inner lipid bilayer. The THB engages the elbow helix, the coupling helix, and the first intracellular loop ICL1 connecting TMH2 and TMH3 in a compact architecture [44]. Interestingly, the rather short ICL1 holds a kinked flexible loop in the middle, exactly at the position where it interacts with the NBD surface. In addition, this loop contains G462 Y463 Y464, which are among the most highly conserved residues shared by all ABCG members including yeast PDRs [9, 16, 62, 91, 92, 93, 94]. Indeed, we show that this loop fulfills an essential role in drug efflux, and in particular in the catalytic cycle, which is triggered after drug and/or ATP binding [17, 62, 95].

We believe that a close proximity of the NBD dimer to the TMD is critical for an efficient coupling of the conformational switch to drug extrusion (Fig. 5). Of note, NBD dimerization is triggered by ATP binding rather than hydrolysis and/or drug trapping in the central cavity. The exact order of events is unclear, since it remains to be shown that the ATP‐free NBDs exist *in vivo* or when the ADP/ATP exchange occurs. Given high cytoplasmic ATP concentrations in the mm range [96, 97], both NBDs may always be decorated by ATP even in the absence of drugs in the central cavity. Indeed, ABCG2 and yeast PDRs can constitutively hydrolyze ATP without drug transport [44, 58, 98] but it remains unclear whether a conformational switch or ADP/ATP exchange occurs without drugs trapped in the central cavity. In this scenario, ABCG2 would consume ATP in a futile cycle but not be able undergo the conformational switch. We speculate and propose that ABCG2 only undergoes a conformational switch when drugs are trapped in the large central cavity or along the polar relay lining the translocation channel [12, 44, 82]. Coupling to the switch can then occur, and we believe that our data show that the THB of the transmission interface, and in particular the GYY loop ICL1, plays an essential role in the coupling of the conformational switch to substrate transport. Indeed, overlaying the cryo‐EM structures of ABCG2 in both inward‐ and outward‐facing states indicates dynamic local movements at the transmission interface. This motion is key for the NBD‐TMD interaction, and requires a flexible element such as ICL1 to support the conformational switch. We reason that the THB enables such a rotational movement by its helices, including the elbow helix at the inner membrane leaflet. Indeed, both elbow helix and ICL1 [44] are clearly engaging in the control of the NBD‐TMD cross talk [44, 69, 99, 100]. Importantly, a salt bridge between two conserved residues, R383 in the elbow helix and E458 of ICL1, stabilizes the transmission interface, which is also critical for ABCG2 biogenesis [44]. ICL1 connects TMH2 and TMH3 and thus forms a structural element that protrudes from the inner membrane leaflet to touch the NBD dimer surface (Fig. 5). To increase the stability, ICL1 is embedded in the THB, making it both flexible and rigid, enabling the control of the conformational switch during the catalytic cycle. Hence, the conserved kinked GYY loop in ICL1 acts as a molecular clutch that enables an efficient coupling to the switch after NBD dimerization. Of note, the ICL1 of ABCG2 is relatively short when compared to other ABC exporters, but that in fact may be necessary to ensure the efficient communication of the THB with the TMD during the coupling step.

The THB holds three helices which seem to engage in close physical interactions. Remarkably, the THB cluster interacts along a highly conserved and shared interface. Strikingly, each THB helix holds at least one residue essential for ABCG2 biogenesis and function. For example, the mutational hot spot helix in ABCG2 carries the Q141K and R147W polymorphisms, both of which are linked to defective urate transport in gout owing to reduced stability of ABCG2 [67, 101]. Further, E458 in ICL1 and R385 in the elbow helix are essential for ABCG2 function [44].

Importantly, mammalian ABCGs and fungal PDRs share the two most highly conserved tyrosine residues in the GYY stretch of ICL1. G462 at the bottom of the ICL1 is important for protein folding. This region requires a kinked structure, as well as a small, flexible residue essential for proper folding of ICL1, since we show that a G462P exchange abrogates proper biogenesis. Y463 turns its side chain into the center space between the hot spot helix and coupling helix of the ICL1. Interestingly, this region shares highly conserved hydrophobic residues, including tryptophan, leucine, methionine, or phenylalanine present in other ABCG family members [41]. Hence, it is tempting to speculate that the resulting hydrophobic interactions may also participate in stabilizing the NBD‐ICL1 interface. While the aromatic ring or hydrophobic side chain of Y463 is not necessary for folding, it is required for full transport function or stability of the NBD‐ICL1 interface. Y464 is the most highly conserved residue of ICL1, with the aromatic ring remaining in the center of the THB in both inward‐ and outward‐facing states of ABCG2. Y464 maintains its position during the conformational switch. Most strikingly, the aromatic ring is not essential for ABCG2 biogenesis, as mutants show normal surface trafficking, but any mutational change destroys efflux function and the catalytic transport cycle. Of note, we believe that Y464 may also engage in a salt bridge to maintain the stability of the transmission interface during drug transport. However, this is difficult or impossible to test, since any mutational change of Y464 abrogates ABCG2 function. Hence, these data demonstrate that position 464 in ICL1 must be occupied by a tyrosine to enable and maintain intramolecular NBD‐TMD interactions and to drive the conformational switch.

To further support our notion about the pivotal importance of Y464 for the catalytic cycle, we inspected putative transmission interfaces in evolutionary distinct ABC proteins. Indeed, several ABCB exporters maintain a NBD‐TMD interface by engaging a tyrosine in a salt bridge interaction between two intracellular helices [12, 28, 30, 32, 102]. In P‐gp, the NBD‐TMD interaction is set via a ‘ball‐and‐socket’ joint [30, 47], in which the ICL or coupling helix, extends from the TMD, and forms a ball‐like structure that docks into the NBD cleft, so as to restrict dynamic NBD movements [30, 47]. The superimposable structures of THB helices imply that ABCG2 has a similar ‘ball‐and‐stick’ formation in the transmission interface.

Although counterintuitive at first glance, the NBD‐TMD interaction via the transmission interface requires ICL1 flexibility but rigidity of the THB. The rigidity may derive from a Y464 salt bridge and the flexibility from a rotational motion of the THB, in particular supported by the elbow helix at the membrane. This concerted movement will contribute to NBD dimer formation, but also bring the NBD surface closer to the TMD. ICL1 is thus acting as the molecular spring, facilitating the NBD ‘lifting’. The THB is the ‘clutch’ that ensures proper coupling to the switch triggering transport. Of note, a conserved kinked loop is also present in the heterodimer ABCG5/G8 and in other ABCG family members, which show a highly restricted cholesterol substrate specificity [9, 91, 92, 103, 104, 105]. These observations strongly suggest that the evolutionary conserved THB is mainly related to the mechanical coupling during the switch rather than to controlling substrate specificity. Interestingly enough, the prokaryotic tripartite antibiotic exporter MacB [106, 107, 108] also contains an amphipathic elbow helix at the inner membrane leaflet. Likewise, MacB harbors a similar THB network at the transmission interface, with a putative salt bridge between E252 in the elbow helix and R554 in the coupling helix, in close proximity of the conserved Y112 residue from the NBD. Hence, the THB may be a conserved architecture in transmission interfaces, facilitating NBD‐TMD interactions in bacterial and eukaryotic ABC proteins.

Finally, upon completion of the conformational switch to the outward drug‐releasing state, the ABCG2 transporter is reset into the inward‐facing ATP‐binding and/or drug‐trapping state by ATP hydrolysis that drives the closed NBD dimer apart before ADP replacement by ATP [48, 57, 61, 68, 109]. Simultaneously, the NBD separation expands the compressed drug‐trapping cavity before a new transport cycle can commence (Fig. 5). Taken together, our results demonstrate that the THB in the ABCG2 transmission interface has a pivotal role in the mechanical coupling of substrate recognition in the central cavity with the conformational switch driving drug expulsion. Hence, these novel insights provide better understanding of dynamic structure–function relationships of ABC proteins, and pave the way for a complete understanding of the ABCG2 catalytic cycle. Since we identify a single residue responsible for the coupling, the results offer therapeutic promises, as a successful targeting of the GYY stretch in ICL1 may help to tackle anticancer resistance caused by ectopic overexpression of ABCG2 in drug‐refractory malignancies.

## Author contributions

NK and KK wrote the manuscript. NK performed all experiments and interpreted the data together with KK.

## Supporting information


**Fig. S1.** Amino acid sequence alignment of mammalian ABCGs and yeast PDRs.
**Fig. S2.** Relative quantification of mature ABCG2 protein by immunoblotting.
**Table S1.** Oligonucleotide primers used to generate ABCG2 mutations.

## Data Availability

The authors confirm that all data underlying the findings are fully available without restrictions. All relevant data are within the paper and the Supporting information.
