# Supplementary material for: The first intracellular loop is essential for the catalytic cycle of the human ABCG2 multidrug resistance transporter
Source: FEBS Lett. 2020 Nov 21;594(23):4059–75. doi: 10.1002/1873-3468.13994 (PMC7756363; doi:10.1002/1873-3468.13994)
Supplement: Supplementary file 1 — Fig. S1. Amino acid sequence alignment of mammalian ABCGs and yeast PDRs. Fig. S2. Relative quantification of mature ABCG2 protein by immunoblotting. Table S1. Oligonucleotide primers used to generate ABCG2 mutations. [file FEB2-594-4059-s001.docx]

**Supplementary Information**

**The First Intracellular Loop is Essential for the Catalytic Cycle of Human ABCG2 Multidrug Resistance Transporter**

Narakorn Khunweeraphong^1,2^ & Karl Kuchler^1,§^

From the

^1^ Medical University of Vienna, Max Perutz Labs Vienna, Center for Medical Biochemistry

Campus Vienna Biocenter, Dr. Bohr-Gasse 9/2, A-1030 Vienna, Austria

^2^ St. Anna Children’s Cancer Research Institute-CCRI, A-1090 Vienna, Austria

^§^ **To whom all correspondence should be addressed:**

Karl Kuchler

Medical University of Vienna, Center for Medical Biochemistry

Max Perutz Labs Vienna, Campus Vienna Biocenter,

A-1030 Vienna, Austria

Phone: +43-1-4277-61807; FAX: +43-1-4277-9618

e-mail: [karl.kuchler@meduniwien.ac.at](javascript:linkTo_UnCryptMailto('kygjrm8iypj,isafjcpYkcbslgugcl,ya,yr');)

**Keywords:** multidrug transporter, anticancer resistance, mechanism, transmission interface, ABC Transporters, ABC catalytic cycle

**Supplementary Figures and Legends**

**
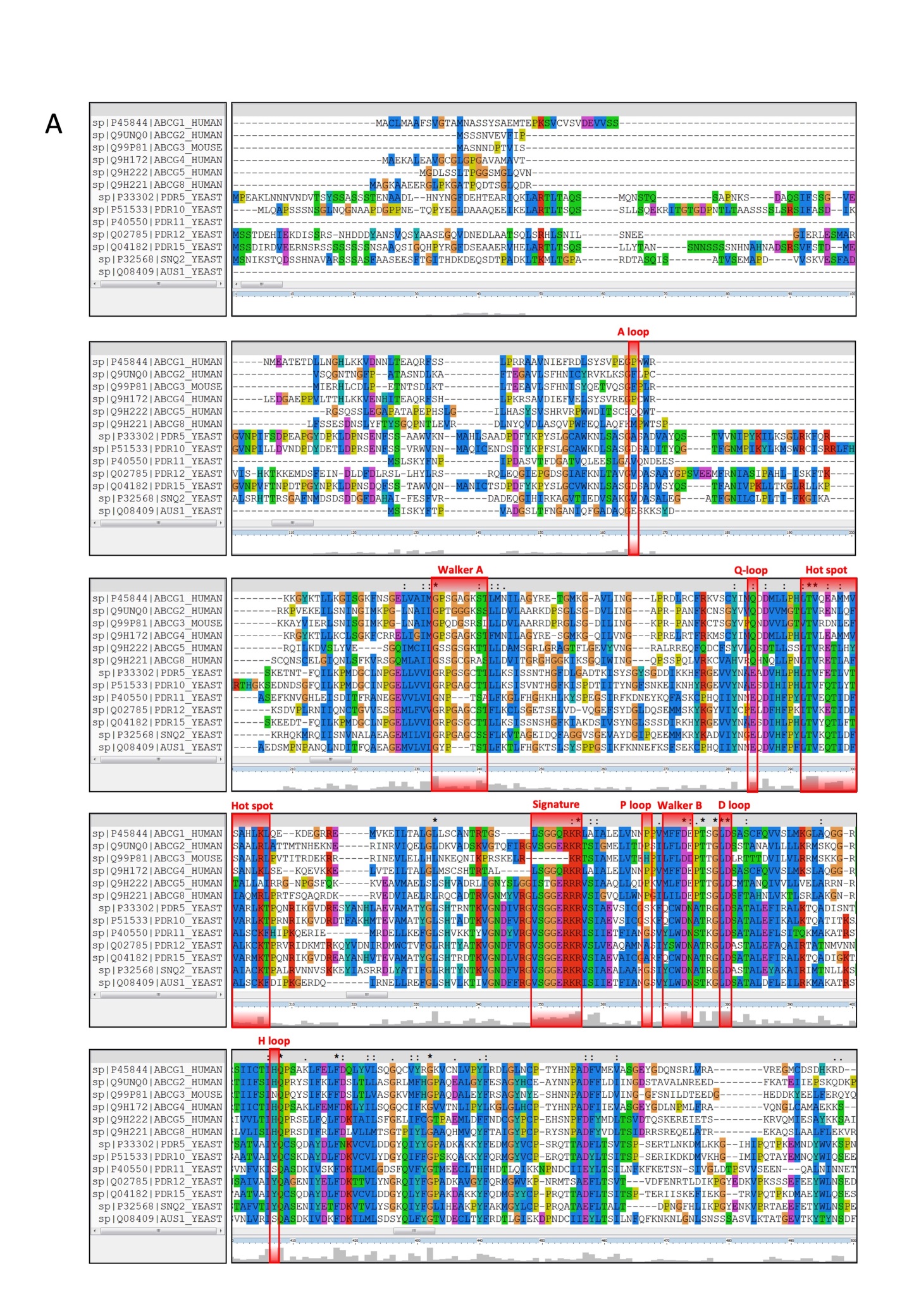
**

**
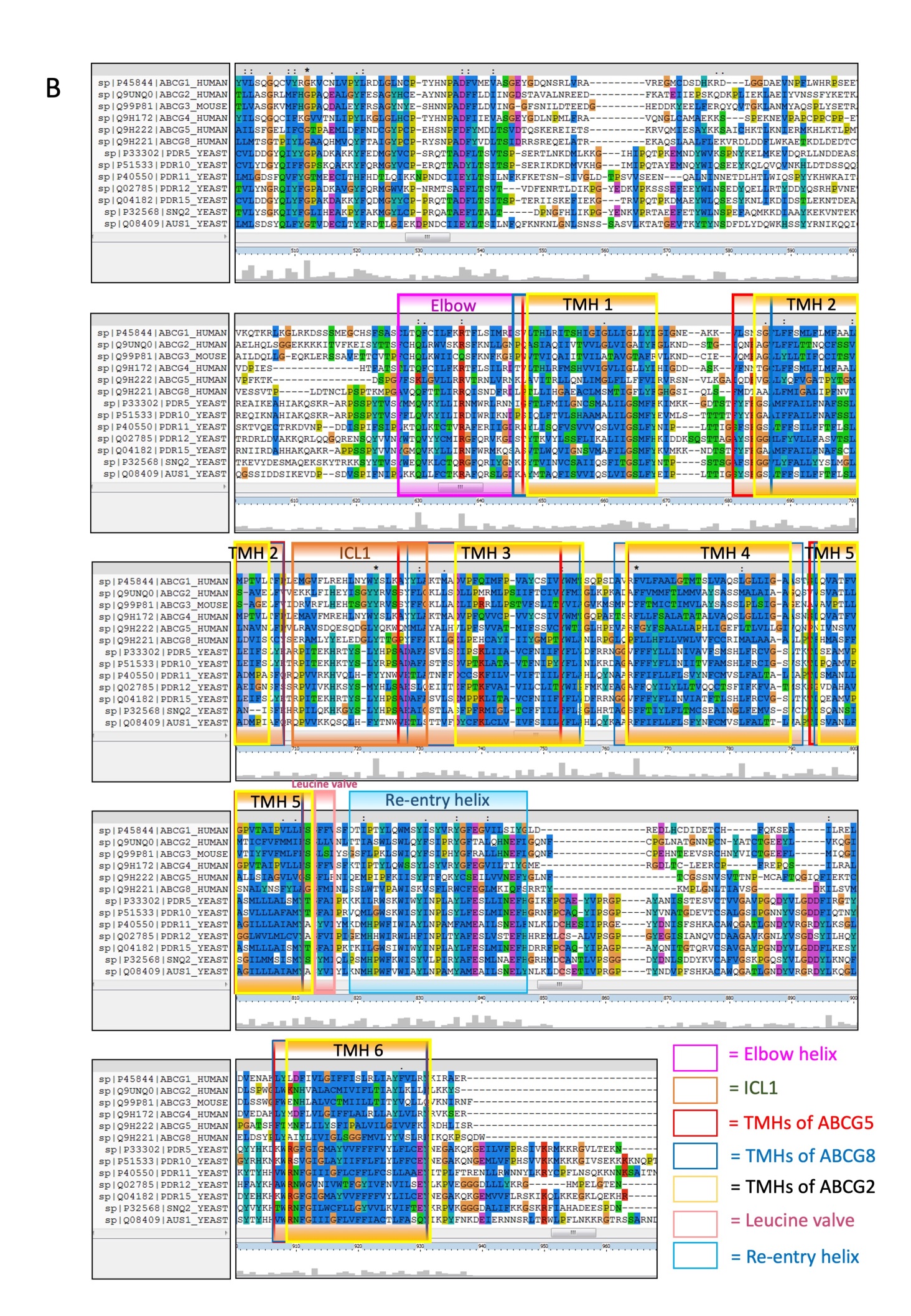
**

**Supplementary Fig. 1. Amino acid sequence alignment of mammalian ABCGs and yeast PDRs.**

The alignments were analyzed by ClustalX2 using primary sequences from mammalian ABCGs and the first half of yeast PDRs proteins. The conserved residues are highlighted and the conservative scores are represented as the height of the grey bar at the bottom of each position.

(A) Alignment of the NBDs shows the conserved regions marked by the red boxes. (B) Alignment of the TMDs shows the conserved regions as indicated in the color-code boxes; elbow helix (pink) and re-entry helix (light blue). The putative transmembrane helices TMH 1-6 are marked based on the structures of ABCG2 (yellow), ABCG5 (red) and ABCG8 (blue), respectively.

**
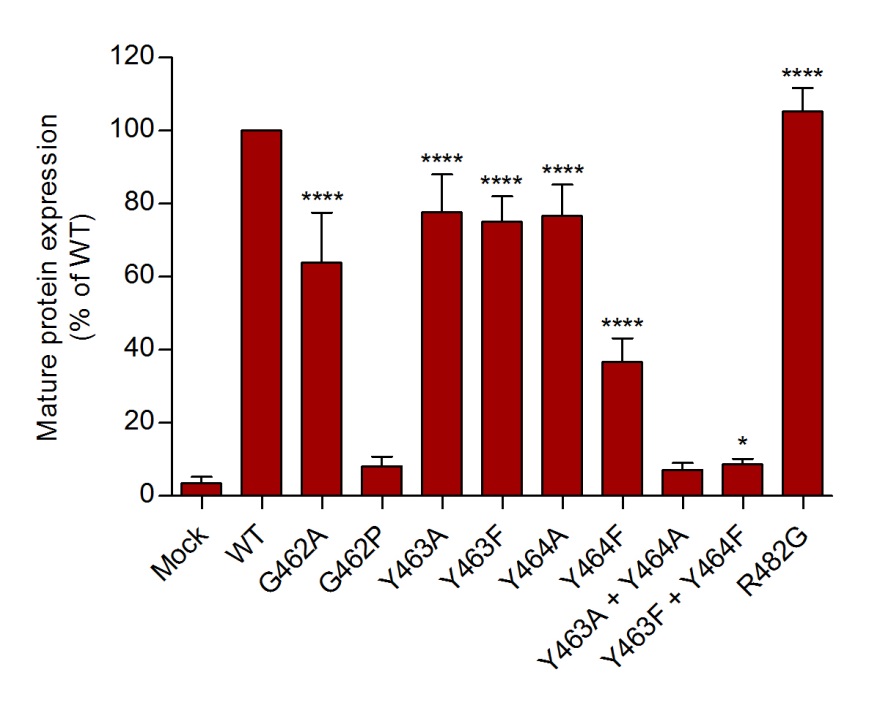
**

**Supplementary Fig. 2. Relative quantification of mature ABCG2 protein by immunoblotting.**

The mature ABCG2 protein levels and β-actin signals on immunoblots were quantified by densitometry. ABCG2 levels were individually normalized to β-actin and data are represented as a percentage relative to WT from several independent experiments (n = 8-10). All data are shown as means + SEM. *****P* < 0.0001, **P* < 0.1 vs. empty plasmid-transfected HEK293 control (mock).

**Supplementary Table: Oligonucleotide primers used to generate ABCG2 mutations**

| **Mutants** | **Forward primer (5'->3')** | **Reverse primer (5'->3')** |
| --- | --- | --- |
| G462A | GAATACATCAGCGcATACTACAGAGTG | CACTCTGTAGTATgCGCTGATGTATTC |
| G462P | GAATACATCAGCccATACTACAGAGTG | CACTCTGTAGTATggGCTGATGTATTC |
| Y463A | CATCAGCGGAgcCTACAGAGTGTC | GACACTCTGTAGgcTCCGCTGATG |
| Y463F | CATCAGCGGATtCTACAGAGTGTC | GACACTCTGTAGaATCCGCTGATG |
| Y464A | CAGCGGATACgcCAGAGTGTCATC | GATGACACTCTGgcGTATCCGCTG |
| Y464F | CAGCGGATACTtCAGAGTGTCATC | GACACTCTGTAGaATCCGCTGATG |
| Y463A + Y464A | GAATACATCAGCGGAgcCgcCAGAGTGTCATCTTATTTC | GAAATAAGATGACACTCTGgcGgcTCCGCTGATGTATTC |
| Y463F + Y464F | CATCAGCGGATtCTtCAGAGTGTCATC | GATGACACTCTGaAGaATCCGCTGATG |
